# A Machine Learning Platform for Isoform-Specific Identification and Profiling of Human Carbonic Anhydrase Inhibitors

**DOI:** 10.3390/ph18071007

**Published:** 2025-07-05

**Authors:** Lisa Piazza, Miriana Di Stefano, Clarissa Poles, Giulia Bononi, Giulio Poli, Gioele Renzi, Salvatore Galati, Antonio Giordano, Marco Macchia, Fabrizio Carta, Claudiu T. Supuran, Tiziano Tuccinardi

**Affiliations:** 1 Department of Pharmacy, University of Pisa, 56126 Pisa, Italy; lisa.piazza@phd.unipi.it (L.P.); miriana.distefano@farm.unipi.it (M.D.S.); giulia.bononi@unipi.it (G.B.); giulio.poli@unipi.it (G.P.); salvatore.galati@farm.unipi.it (S.G.); marco.macchia@unipi.it (M.M.); 2 Telethon Institute of Genetics and Medicine, 80078 Naples, Italy; c.poles@tigem.it; 3 Genomics and Experimental Medicine Program, Scuola Superiore Meridionale (SSM, School of Advanced Studies), 80138 Naples, Italy; 4 Department of Neurofarba, Section of Pharmaceutical and Nutraceutical Sciences, University of Florence, Polo Scientifico, Via U. Schiff 6, Sesto Fiorentino, 50019 Firenze, Italy; gioele.renzi@unifi.it (G.R.); fabrizio.carta@unifi.it (F.C.); claudiu.supuran@unifi.it (C.T.S.); 5 Sbarro Institute for Cancer Research and Molecular Medicine, Center for Biotechnology, College of Science and Technology, Temple University, Philadelphia, PA 19122, USA; antonio.giordano@temple.edu; 6 Consorzio Interuniversitario Nazionale per la Scienza e Tecnologia dei Materiali (INSTM), 50121 Firenze, Italy

**Keywords:** carbonic anhydrase, isoform selectivity, machine learning, virtual screening, SHAP, drug discovery

## Abstract

**Background/Objectives:** Human carbonic anhydrases (hCAs) are metalloenzymes involved in essential physiological processes, and their selective inhibition holds therapeutic potential across a wide range of disorders. However, the high degree of structural similarity among isoforms poses a significant challenge for the design of selective inhibitors. In this work, we present a machine learning (ML)-based platform for the isoform-specific prediction and profiling of small molecules targeting hCA I, II, IX, and XII. **Methods:** By integrating four molecular representations with four ML algorithms, we built 64 classification models, each extensively optimized and validated. The best-performing models for each isoform were applied in a virtual screening campaign for ~2 million compounds. **Results:** Following a multi-step refinement process, 12 candidates were identified, purchased, and experimentally tested. Several compounds showed potent inhibitory activity in the nanomolar to submicromolar range, with selectivity profiles across the isoforms. To gain mechanistic insights, SHAP-based feature importance analysis and molecular docking supported by molecular dynamics simulations were employed, highlighting the structural determinants of the predicted activity. **Conclusions:** This study demonstrates the effectiveness of integrating ML, cheminformatics, and experimental validation to accelerate the discovery of selective carbonic anhydrase inhibitors and provides a generalizable framework for activity profiling across enzyme isoforms.

## 1. Introduction

Carbonic anhydrases (CAs) are a family of ubiquitously expressed metalloenzymes which catalyze the interconversion between carbon dioxide (CO_2_) and water (H_2_O) into bicarbonate ions (HCO_3_^−^) and protons (H^+^). In light of the essential role that this reaction plays in sustaining life, CAs exhibit a widespread occurrence across multiple biological kingdoms. To date, eight unrelated gene families have been recognized as being responsible for encoding the corresponding families of enzymes: α, β, γ, δ, ζ, η, θ, and ι, with their distribution varying within the phylogenetic tree [1]. Specifically, only the α family is expressed in humans. It comprises 16 distinct CA isoforms with different cellular and tissue localization, but only 12 of them show catalytic activity. Human CAs (hCAs) are involved in a multitude of physiological functions (pH homeostasis, electrolyte secretion, respiration), biosynthetic pathways (lipogenesis, gluconeogenesis), and biological processes (calcification, bone resorption). Their pivotal role in this biochemical functions’ repertoire underpins their connection with several pathological conditions, including inflammation, obesity, glaucoma, epilepsy, and cancer. Consequently, hCAs consistently attract significant interest as therapeutic targets, and their modulators are regarded as promising candidates for a broad spectrum of clinical applications [2].

Nevertheless, the widespread expression across different tissues combined with the high degree of sequence and structural similarity of hCA isoforms pose a significant challenge in drug development, particularly in achieving selective inhibition. In this scenario, profiling the inhibitory activity of compounds across different hCA isoforms can shed light on their modulatory characteristics, thereby facilitating a deeper understanding of their selectivity. This valuable insight holds promise for the identification of selective inhibitors with high therapeutic potential and represents a valuable starting point for more effective treatments.

To address the inherent challenges of high-throughput screening and molecule profiling in drug discovery, computer-aided drug design (CADD) approaches have proven to be valuable strategies for identifying the most promising hits prior to biological assays. Several studies in the literature have demonstrated the successful application of computational methods targeting CAs using structure-based approaches [3,4,5,6]. However, these methods are inherently limited by the availability of crystallographic data to guide the rational design of inhibitors by exploiting structural insights. In contrast, ligand-based techniques do not rely on 3D structural information and, thanks to the extensive data available, were effectively applied to shed light on structure–selectivity relationship for carbonic anhydrases. It is exactly the increasing availability of data, coupled with the technological advancements of recent years, that has ushered in a new frontier in drug discovery: machine learning (ML). Recent literature is replete with examples of ML applications, which have led to the identification of promising compounds and have significantly advanced our understanding of molecular activity across diverse targets [7,8,9,10,11].

Nonetheless, despite the widespread application of artificial intelligence in drug discovery, the use of ML to target CAs still presents significant opportunities for improvement. To date, only a limited number of studies have employed ML to specifically target hCAs, although with different objectives and applications. Over time, efforts have been undertaken to broaden the scope. Indeed, ML models developed by Kırboğa and Işık, and those reported by Kim and co-workers targeted two isoforms, specifically hCA I and hCA II [12,13]. The former study was purely computational and investigated the impact of small structural components in the inhibitory activity and potency of molecules. The latter used the developed models to identify dual inhibitors of kinases and CAs for anticancer therapy, despite the principal isoforms implicated in tumorigenesis being hCA IX and hCA XII. The work of Galati and co-workers addressed the challenges associated with oncological treatments involving hCAs by developing an ML model designed to predict selectivity for hCA IX over hCA II, offering a more targeted approach for the development of selective inhibitors [14]. Tinivella and collaborators, on the other hand, developed ML models capable of predicting activity against three isoforms: hCA II, hCA IX, and hCA XII [15].

Overall, ML has yet to be fully leveraged for the identification of novel hCA inhibitors or for profiling the potential inhibitory activity of compounds across a broader and more relevant spectrum of isoforms. In this study, we harnessed the power of ML to enhance precision targeting and streamline the drug discovery process. With the aim of identifying new promising hCA inhibitors, we developed an ML-based tool to profile the potential inhibitory activity of molecules against four hCA isoforms of interest: hCA I, hCA II, hCA IX, and hCA XII. The former two isozymes are cytosolic forms primarily associated with conditions such as glaucoma, epilepsy, and neuropathic pain; the latter are instead membrane-bound enzymes predominantly linked to tumor development and inflammation [2,16]. To enhance target profiling and go beyond conventional virtual screening (VS) studies, we employed an ML-based strategy that integrates insights from the literature. Specifically, we identified compounds published in previous studies that had not been reported as CA inhibitors and assessed their potential activity against the selected isoforms. This approach led to the identification of 12 candidate compounds, which were subsequently experimentally tested, revealing detailed activity profiles and, in some cases, inhibitory effects with *K*_i_ values in the low nanomolar to sub-micromolar range. These findings highlight the effectiveness of our profiling protocol in selecting promising hCA inhibitors, demonstrating its ability to identify compounds with valuable and desirable properties, and paving the way for future hit-to-lead optimization studies. Furthermore, our approach provides relevant insights into the context of off-target activity prediction, which represents an essential aspect of drug discovery aimed at minimizing adverse effects and optimizing therapeutic efficacy. The prediction of target selectivity and cross-reactivity offers a twofold advantage: guiding the design of more selective inhibitors and uncovering potential drug repurposing opportunities. By exploiting ML, our platform serves as a cost-effective and resource-efficient prediction tool for target profiling.

**Figure 1:**
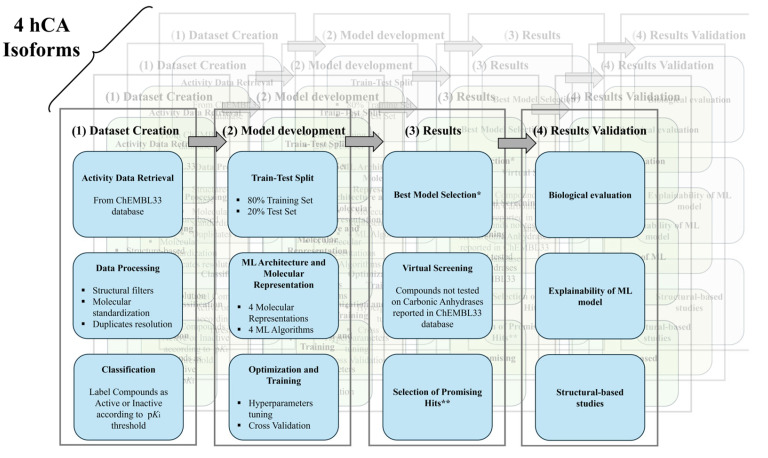
Schematic diagram that summarizes the entire workflow. * For hCA I and II, the best model selected was SVM-Morgan FPs; for hCA IX and XII, it was RF-Morgan FPs. ** Predicted active compounds for hCA I, II, IX, and XII (55,678, 14,050, 7933, and 15,896, respectively) were subjected to medicinal chemistry and toxicity filters.

The overall workflow of our study, including the step-by-step procedures followed, is illustrated in Figure 1.

## 2. Results

### 2.1. Data Retrieval and Datasets Inspection

With the aim of developing a predictive ML platform to gain insights into the potential inhibitory activity of compounds against a significant spectrum of hCA isoforms, we targeted hCA I, hCA II, hCA IX, and hCA XII. In addition to the clinical significance of these isoforms, their selection was guided by the availability of sufficient data for the development of robust ML models. ChEMBL 33 [17] was selected as a source database for activity data retrieval, and compounds with reported inhibitory activity on the targeted isoforms were retrieved and subjected to a thorough data filtering procedure (see Section 4) to obtain four high-quality datasets, one for each isozyme. Indeed, the quality of the dataset employed to develop an ML model significantly influences its performance [18]. Thus, a thorough and comprehensive data processing protocol lays the groundwork for a robust pipeline.

Based on the p*K*_i_ value distribution, for each dataset, we identified a proper activity threshold for binary classification, and we labeled compounds accordingly. In detail, all compounds exhibiting an inhibitory activity, expressed as p*K*_i_, higher than the selected cutoff were labeled as *active*, whereas those with lower values were labeled as *inactive*. To reduce the impact of a strict classification cutoff, molecules with a reported p*K*_i_ within ±0.25 of the selected threshold were excluded from the corresponding dataset. These compounds were indeed more prone to classification ambiguity, as minor fluctuations in enzymatic activity near the threshold can lead to label shifting. A final integration of inactive compounds was carried out. These compounds, identified by a “>” in their standard relation type, were confidently classified as inactive based on their exceeding the defined threshold. This refinement produced the definitive datasets, which were then split into training and test sets. The number of compounds in the four datasets is reported in Section 4. Each dataset contains over 2000 training compounds, which is especially advantageous for developing ML models. Indeed, the large volume of data provides the model with a sufficient number of examples to learn statistical patterns, improving its ability to make accurate predictions. The largest dataset is the one collected for hCA II which comprises a total of about 5000 training and test compounds. Moreover, the proportion of active and inactive compounds is the same across each training/test set pair. This ensures that the models will be tested in a consistent manner, preventing any potential bias arising from differences in dataset composition. Additionally, to gain deeper insight into the chemical space covered by our datasets, we employed the t-distributed stochastic neighbor embedding (t-SNE) [19] technique to specifically assess the alignment between the chemical space of each training set and the corresponding test set. Upon results evaluation (Figure S1 in Supporting Information), we could confirm consistency between the chemical space of each isoform-specific train/test set pair, therefore ensuring that models were trained and evaluated on comparable structural spaces.

### 2.2. Models Development and Evaluation

The datasets created were used to develop binary classifier models to discriminate between compounds potentially active as inhibitors of a specific isoform and those lacking this potential. For each targeted isozyme, we combined four ML algorithms, namely Support Vector Machine (SVM), Random Forest (RF), K-Nearest Neighbors (KNN), and Gaussian Process (GP), with four molecular representations—Morgan fingerprints (FPs), PubChem FPs, RDKit FPs, and RDKit descriptors—resulting in 16 ML models developed for each isoform.

It is worth mentioning that values related to molecular descriptors exist within different ranges and magnitudes. Without proper mathematical adjustment, descriptors with larger values may disproportionately influence the training phase, potentially leading to biased models that fail to capture the contribution of smaller-scale features. To address this issue, descriptors’ values were normalized to ensure a balanced contribution of all the features.

All the generated models were subjected to an exhaustive hyperparameter optimization to find the suitable setup for achieving the best performance. Subsequently, each model was evaluated using a 10-fold cross-validation (CV) protocol to assess its predictive capability and robustness across different data partitions (see Section 4 for details). For this purpose, the Matthews Correlation Coefficient (MCC) [20] was used as a scoring metric. Although the results achieved by each model during this 10-fold CV (Table S1) demonstrate overall robustness across all the analyzed models, some noticeable trends can still be observed. The molecular representation generally associated with the least satisfactory performance was RDKit descriptors, whereas fingerprint-based representations yielded higher performance, with the Morgan FPs being the best one in most cases. From an algorithmic perspective, SVM- and RF-based models emerged as the best-performing methods, while KNN-based ones appeared as the least effective in this case study. An interesting trend can also be noticed in the models related to hCA I and II, which achieved slightly higher performance compared to those related to isoforms IX and XII. This difference could likely be attributable to the larger amount of data available in the datasets for the former models.

Upon hyperparameter tuning and cross validation, the optimal arrangement for each model was selected and applied to train the model on the corresponding isoform-specific training set. The 64 fully developed models were then subjected to an in-depth evaluation using the isoform-specific hold-out test set. This step assessed their performance on unseen data, simulating a real-world scenario where the models are used to screen compounds with unknown activity against the target isoforms. For an exhaustive assessment, all models were evaluated using six different metrics (see Section 4.4): MCC, Precision, Recall, Specificity, Accuracy, and Negative Predictive Value (NPV). These benchmarks provide a comprehensive insight into the models’ predictive performance, especially regarding their potential employment in a vs. protocol. Precision quantifies the exactness of positive predictions, while Recall reflects the ability to identify active molecules within the total set of active ligands present in the dataset. High Precision is crucial for VS, as only a small subset of compounds from the screened library are ultimately selected for experimental evaluation. However, focusing solely on Precision can lead to a high number of false negatives, potentially excluding promising candidates. For this reason, Recall is equally important in VS, as it minimizes the risk of losing active molecules during the screening process, ensuring a comprehensive identification of hits for further optimization. It therefore becomes essential to evaluate models using multiple metrics, with each assessing different aspects of the performance, to gain a comprehensive understanding of their effectiveness. Based on the scores achieved, the top-performing model was identified for each isoform. Their detailed performance profile is reported in Table 1.

The results obtained clearly showcase the impact of molecular representation on the effectiveness of predictions. Specifically, in this case study, Morgan FPs emerged as the representation that allowed the models to most efficiently detect patterns for successful learning. Instead, from the algorithm perspective, the ones that proved to be a major success were SVM and RF, being the most effective for hCA I-II and IX-XII, respectively. Additionally, the top-scoring models for hCA I-II proved to achieve slightly better performance than the ones for isoforms IX and XII. These findings are in accordance with the trends previously detected during the CV results analysis. Nonetheless, all four models achieved high performance, with a correct prediction rate always above 85%, and an MCC above 0.70. This overview emphasizes the models’ robustness and their capacity to accurately distinguish between active and inactive compounds, thereby supporting their use in the subsequent vs. protocol.

### 2.3. Virtual Screening and Results Analysis

Considering the promising results achieved, the top-performing model for each isoform (reported in Table 1) was employed in a vs. protocol to identify molecules with hCA inhibition activity, while also providing a potential inhibitory activity profile against the considered isoforms. From the ChEMBL 33 [17] database, we obtained a curated library of around 2 million compounds (see Section 4.6), which was employed to carry out four parallel vs. procedures, each driven by the top-performing model identified for a specific hCA isoform. From each vs. pipeline, the compounds predicted as active with a probability score ≥ 50% were retained for further refinement.

Specifically, for hCA I, hCA II, hCA IX, and hCA XII, this corresponded to 55,678, 14,050, 7933, and 15,896 compounds, respectively. To ensure the selection of a small set of high-quality, synthetically feasible, and pharmacologically relevant compounds, we implemented a multi-step filtering pipeline based on established medicinal chemistry principles and toxicity considerations. First, all predicted active compounds were pooled and filtered to retain only commercially available molecules. We then applied Pan-Assay Interference (PAINS) filters [21] by using MolBook UNIPI version 1.6 [22] to remove promiscuous compounds potentially exhibiting non-specific bioactivity in assays, reducing the dataset to a total of 13,879 molecules. Next, we focused on the pharmacological suitability of compounds and their synthetic accessibility, to ensure that the selected compounds were not only theoretically promising but also feasible to synthesize in a laboratory setting. We therefore applied Lipinski’s rule of five and Veber’s rule to prioritize compounds with high drug-likeness. The resulting subset, which consisted of 9990 molecules, was further refined to eliminate chemically undesirable substructures and improve safety profiles. We employed multiple structural alert filters—including Brenk alerts, Bristol-Myers Squibb (BMS) alerts, Inpharmatica alerts, and Novartis NIBR rules [23,24,25]—all of which identify potentially reactive, unstable, or toxicophoric substructures, thereby reducing the number of promising compounds to 7100 molecules. An additional toxicity filter was conducted using VenomPred 2.0 platform [26], a powerful predictive tool that enables accurate and early identification of potential toxicity risks. Specifically, we focused on four toxicity endpoints deemed relevant in the context of hCA inhibitor development: eye irritation, acute oral toxicity, carcinogenicity, and hepatotoxicity. hCA inhibitors are used to treat glaucoma, making it essential to avoid ocular irritation. When administered orally for systemic effects, assessing acute oral toxicity is also crucial. Additionally, since hCA IX and hCA XII are anticancer targets, it is important to ensure that the selected compounds do not exhibit unintended carcinogenic effects. Lastly, hepatotoxicity screening is important, as hCAs play a key role in ammonia detoxification. In patients with liver dysfunction, hCA inhibition can exacerbate ammonia accumulation, increasing the risk of hepatic encephalopathy [27]. Therefore, minimizing hepatotoxicity is essential for the safe development of novel inhibitors. By removing compounds with a relevant predicted toxicity potential, we obtained a subset of 3879 compounds. With the aim of maintaining a high degree of structural diversity and uncovering potential off-target interactions or new pharmacological activities, we focused on molecules with reported activity in the literature against targets other than CAs. Next, we excluded compounds that exhibited significant structural similarity to those in the training set. Using PubChem FP representations, each predicted active molecule was compared to the isoform-specific training set, and compounds with a Tanimoto score greater than 0.80 were removed. As a result of our filtering pipeline, we obtained 380, 47, 30, and 35 compounds for hCA I, hCA II, hCA IX, and hCA XII, respectively. These compounds were then ranked based on their probability score, and consequently, we obtained four refined sets of highly promising compounds, each tailored to a specific hCA isoform and selected for their potential inhibitory activity. Finally, to identify potential multi-target inhibitors, we cross-referenced the datasets to account for compounds predicted to be active against multiple hCA isoforms.

This approach allowed us to obtain 12 molecules exhibiting a comprehensive predicted inhibitory activity profile across all four isoforms. These molecules were ultimately purchased and subjected to enzymatic assays to assess their activity on the targeted isoforms (see Section 4.7 for details). The experimental results obtained are reported in Table 2.

VS is a well-established method for identifying promising compounds for further optimization. While sub-micromolar activity is not strictly required for vs. hits, it still represents a desirable threshold indicative of high potential [28]. To demonstrate the robustness of the proposed approach, we intentionally adopted 1 µM as a cutoff to classify the experimental data, enabling a clear and meaningful comparison between predictions and experimental results. Specifically, a molecule was considered as biologically relevant if it showed an experimental *K*_i_ lower than 1 µM. The analysis of experimental activity compared with the ML platform predictions (Table S2) yielded an overall hit rate of 77%, with 37 out of 48 predictions being in accordance with experimental data. In detail, 24 experimental assays confirmed the predicted activity, while 13 outcomes were correctly identified as inactive. Conversely, 11 predictions deviated from the experimental data, with 9 out of 11 being false positives. Notably, only two false negatives were observed, which is favorable in the context of off-target prediction, where minimizing the loss of potential activities is crucial to ensuring that promising compounds are properly evaluated in the early stages of drug development.

Out of the 12 compounds tested, 8 contained a primary sulfonamide moiety (compounds **1**, **2**, **4**, **7**, **8**, **9**, **11**, and **12**). This functional group is widely recognized for its critical role in the inhibition mechanism of CAs. Consistently with its known biological significance, the majority of these sulfonamide-containing compounds demonstrated biological activity below the 1 µM threshold, indicating their high potency. Furthermore, most of these active compounds were accurately predicted as such by the models, highlighting their effectiveness in identifying molecules with this key functional group. Compounds **8** and **12** were predicted to be active against hCA I, but the values of their experimental *K*_i_ (1204.8 nM and 1076.3 nM, respectively) were found to be only slightly above 1 µM, making the predictions incorrect but still borderline, as the biological activities of these compounds remain relevant. Interestingly, compound **12** has previously shown promising inhibitory activity against α-amylase [29], representing a compelling strategy to treat metabolic disorders involving high levels of blood glucose, such as type II diabetes and obesity. In addition, compound **12** was found to reduce the rate of polymerization of the Z mutant (Glu342Lys) of α_1_-antitrypsin [30], a mutation linked to liver diseases such as hepatitis, cirrhosis, and hepatocellular carcinoma. Moreover, Brvar and co-workers reported compound **12** as a bacterial DNA gyrase B inhibitor with promising antimicrobial activity [31]. Thus, our study expanded the knowledge of compound **12** by demonstrating its inhibitory activity against four hCA isoforms, further reinforcing its potential polypharmacological profile and broad therapeutic relevance. In contrast, all compounds lacking the primary sulfonamide moiety were confirmed to be biologically inactive at the 1 µM threshold.

In most cases, the ML models correctly predicted these compounds, with only 5 predictions out of 16 being false positives. It is also noteworthy that our ML platform correctly identified potent inhibitors with activity in the nanomolar range against all four isoforms (i.e., compounds **1**, **9**, and **11**), demonstrating the robustness of the proposed approach. The therapeutic potential of compounds **1** and **11** was further expanded by their previous exploration as possible antimicrobial agents. Specifically, the former was tested for its ability to inhibit biofilm formation in *S. aureus* [32], whereas the latter was investigated for its potential to inhibit DNA helicase in *B. anthracis* and *S. aureus* [33]. Besides potency though, selectivity is another key factor in medicinal chemistry as it contributes to the development of targeted treatments with reduced side effects. Although only about 60% of the predictions for compounds **7** and **8** were correct (five predictions out of a total of eight), the experimental data unveiled the selective inhibition achieved by these compounds. The inhibitory activity of both compounds against hCA II was indeed at least 25-fold higher than the one observed against the other isozymes. Both compounds **7** and **8,** together with **6** and **9**, were investigated for their antimalarial activity in two different screening campaigns [34,35], suggesting their possible optimization for multi-target applications.

Also, compound **4**, previously studied for the potential inhibition of thromboxane synthetase [36], showed notable nanomolar inhibitory activity against the hCA II isoform, but its selectivity toward this isozyme was borderline, being only approximately five times more active against this target than against hCA I. Nevertheless, its hCA I/II inhibitory potency was considerably more compared to its activity against the other two isoforms (hCA IX/XII). It is also interesting to notice that compound **6**, which lacks the primary sulfonamide group, exhibited a less potent but still selective inhibition of hCA II, with *K*_i_ values of 7050.3 nM and above 100 µM for hCA II and the other three isoforms, respectively. Conversely, compound **2**, initially studied for β-secretase inhibition [37], has shown more pronounced activity towards hCA II/XII compared to hCA I/IX. hCA II and XII are implicated in different disease contexts, highlighting the potential therapeutic relevance of compound **2** in targeting a broader spectrum of conditions associated with distinct CA isoforms. In general, the ML platform developed in this study proved to be effective in identifying promising hits with desirable activity profiles and enabled the detection of compounds with significant potential for further optimization and multifaceted therapeutic applications.

### 2.4. Binding Mode Analysis of Compound ***1***

With the aim of suggesting a binding mode in hCAs for compound **1**, which showed the most interesting inhibitory activity among the panel of tested compounds, and analyzing the ligand–protein interaction features that could be at the basis of its selectivity profile, molecular modeling studies including molecular dynamics (MD) simulations and ensemble docking evaluations were carried out. In particular, starting from an X-ray structure of each hCA isoform employed in this study, 100 ns of MD simulation in explicit water environment was performed for each enzyme in order to account for protein flexibility in our binding pose prediction analysis, and thus overcome the typical limitations of docking-based approaches (see Section 4 for details). By using Gold software version 5.1, compound **1** was then docked into 100 different conformations of each hCA isoform, which were derived from the corresponding MD simulations, for a total of 400 different docking evaluations. The 100 different ligand–protein complexes obtained for each hCA isoform as a result of our ensemble approach were then clustered based on the ligand disposition within the protein binding site, and the best cluster of solutions obtained for each CA isoform was selected as the final binding mode of the ligand. By following this approach, we were able to consider the conformational dynamism of both the receptor and the ligand and exploit this feature to better rationalize the selectivity profile of compound **1**. Moreover, the binding mode predicted for the ligand in each hCA isoform was then subjected to an additional energy minimization in an explicit water environment to further refine the conformation of both ligand and protein, and to account for eventual water-mediated ligand–protein interactions often neglected (or only implicitly considered) in classic docking-based approaches (see Section 4 for details).

**Figure 2:**
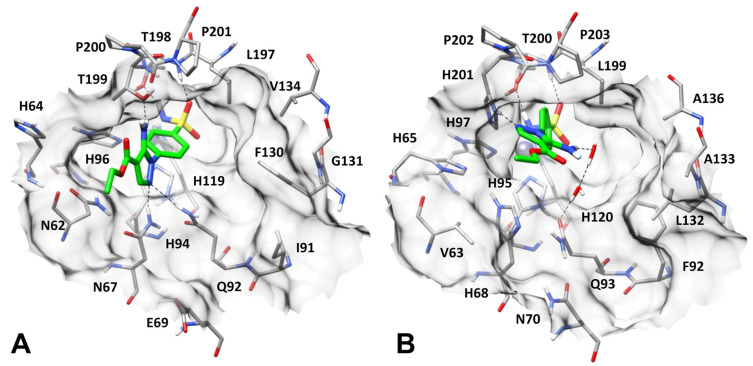
Predicted binding mode of compound **1** in hCA II (**A**) and I (**B**). In each panel, the ligand is shown in green, the binding site residues are displayed in gray, the surface of the binding cavity is shown in transparency, and H-bonds are displayed as black dashed lines.

**Figure 3:**
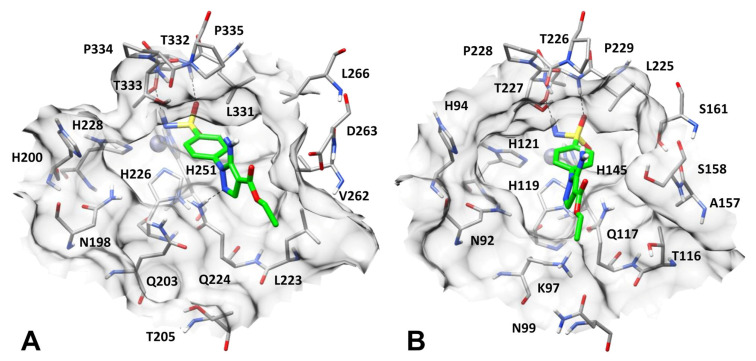
Predicted binding mode of compound **1** in hCA IX (**A**) and XII (**B**). In each panel, the ligand is shown in green, the binding site residues are displayed in gray, the surface of the binding cavity is shown in transparency, and H-bonds are displayed as black dashed lines.

Figure 2 and Figure 3 show the final, energy-minimized structures of hCA I, II, IX, and XII in complex with compound **1** obtained by applying this protocol. As shown in the figures, the sulfonamide group of the ligand is able to form the same pattern of interactions with all four hCA isoforms. In particular, the ligand properly coordinates the prosthetic zinc ion of each protein and forms two different H-bonds with a threonine residue placed in its close proximity, i.e., T198 in hCA II (Figure 2A), T200 in hCA I (Figure 2B), T332 in hCA IX (Figure 3A), and T226 in hCA XII (Figure 3B). Nevertheless, the aminopyrazole ring of compound **1** was predicted to form different interactions in the different enzyme isoforms.

In hCA II, the ligand is placed toward the hydrophilic side of the protein binding site and is able to establish three different H-bonds: two H-bonds with the side chains of N67 and Q92, and an additional one with the hydroxyl group of T199 (Figure 2A). Such network of interactions is believed to strongly anchor the ligand to the hCA II catalytic site, which is in agreement with the low nanomolar inhibitory activity of the ligand against this isoform. Moreover, the phenyl ring of the ligand shows aromatic interactions with the zinc-coordinating H94.

In hCA I, the ligand cannot maintain the same binding mode assumed in hCA II, due to a series of non-conserved residues in the two isoforms. In fact, N67 and N62 present in hCA II are, respectively, replaced by H68 and V63 in hCA I; this change considerably reshapes the lipophilic side of the binding site, which pushes the diarylic core of the ligand toward P202 (Figure 2B). Moreover, the presence of H201 in place of T199 as in hCA II determines a rotation of the ligand pyrazole ring, compared to its binding mode in hCA II, which allows compound **1** to form an H-bond with the side chain of H201. Although by assuming this disposition the inhibitor can only form a water-mediated H-bond network with Q93 (homolog of Q92 in hCA II), its phenyl ring establishes strong aromatic and hydrophobic interactions with H201 and L199, respectively, and further lipophilic contacts are observed between the ligand pyrazole ring and P203 (Figure 2B). Such water-mediated and aromatic/hydrophobic interactions predicted in hCA I are believed to partially compensate for the loss of the two direct H-bonds with N67 and Q92 observed in the hCA II binding site, which would justify the modest decrease in the inhibitory activity of the compound (an approximately 5-fold reduction) against hCA I compared to hCA II.

While in hCA II, compound **1** is disposed of on the lipophilic side of the binding site, in hCA IX, the ligand is placed on the opposite side of the catalytic pocket (Figure 3A). In fact, the longer side chain of Q203 in hCA IX compared to N67 of hCA II shrinks the excluded volume of the binding pocket at its hydrophobic side, which is thus less accessible to the ligand. In contrast, the presence of V262 in place of F130 creates a cleft on its hydrophilic side, delimited by L223, Q224 and V262, where the terminal portion of the ligand is thus predicted to be located. In this disposition, the aminopyrazole moiety of compound **1** can only form a single H-bond with the side chain of Q224 (homolog of Q92 of hCA II) and does not show relevant hydrophobic interactions with the surrounding residues, while the ligand phenyl ring takes primarily lipophilic contacts only with L331 (Figure 3A). The lack of two out of the three H-bonds predicted to be formed by the ligand in hCA II, and the absence of any other strong interaction able to compensate for such loss, is in agreement with the almost 50-fold drop in inhibitory activity observed for compound **1** against hCA IX compared to hCA II.

Finally, in hCA XII, the ligand is oriented at the center of the binding site, with its pyrazole ring placed in a sort of subpocket formed by N92, K97, Q117 and H119, and is unable to form any of the H-bonds predicted in hCA II (Figure 3B). In fact, N67 of hCA II is replaced by K97, whose side chain is bent over N99 to form a charge-assisted H-bond, while the side chain of Q117 (homolog of Q92 of hCA II) is oriented toward A157 (homolog of F130 of hCA II). Moreover, being far from T227 (homolog of T199 of hCA II), the ligand is unable to interact with this residue. Besides the zinc-coordination and the H-bonds formed by its sulphonamide group, which are predicted in all studied hCA isoforms, the most relevant interactions showed by compound **1** in hCA XII correspond to aromatic interactions with H119 formed by its phenyl ring (Figure 3B). These results may justify the over 500-fold lower inhibitory activity determined for the ligand against hCA XII compared to hCA II.

### 2.5. Machine Learning-Based Feature Importance Analysis of Compound ***1***

Given the importance of making ML predictions interpretable, we employed an automated approach to identify the molecular features that most significantly contributed to the predicted activity of compound **1**. Feature importance was assessed using the Shapley value framework, a game theory-based method widely adopted to quantify the contribution of individual features to the output of ML models. Specifically, we applied the SHAP (SHapley Additive exPlanations) [38] approach to evaluate the impact of each fingerprint (FP) bit on the prediction generated for compound **1**. To translate these contributions into structural insights, we adopted a retro-mapping strategy tailored for Morgan FPs, enabling the identification of the chemical substructures responsible for the predicted activity. Each atom was then assigned a weight reflecting its contribution to the prediction, calculated as the sum of the SHAP values of the features it participates in. This sum was normalized by both the number of atoms in each feature and the number of times the atom appeared across relevant features in the molecule. Lastly, RDKit’s mapping functionalities were employed to visualize the magnitude of atomic contributions, allowing us to highlight the molecular regions most influential in the prediction.

**Figure 4:**
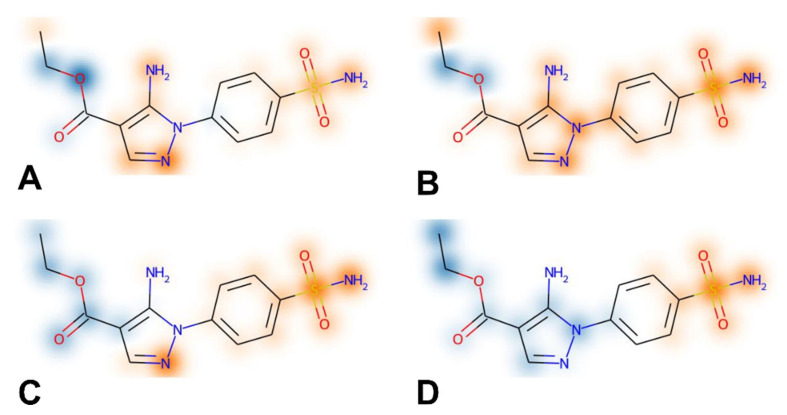
Results obtained from the SHAP analysis for compound **1** related to its prediction of activity against hCA II (**A**), I (**B**), IX (**C**), and XII (**D**). The orange-colored moieties indicate a positive impact on the prediction, whereas blue-colored moieties indicate a negative one.

The feature importance analysis performed by using this approach for compound **1** was consistent with the binding mode predicted for compound **1** in the different hCA isoforms herein studied. In fact, the SHAP evaluation highlighted the sulphonamide group of the ligand as a fundamental moiety for determining the inhibitory activity against all hCA isoforms, while suggesting a different importance of the aminopyrazole moiety for the binding to the different enzymes. In particular, for hCA II, the SHAP analysis highlighted the NH2 group and the unsubstituted nitrogen of the pyrazole ring as two different key portions of the aminopyrazole moiety for determining the activity of compound **1** (Figure 4A), which is consistent with the multiple H-bonds that are predicted to be formed by these moieties of the ligand (Figure 2A). In contrast, the SHAP evaluation suggests the overall importance of the whole aminopyrazole ring for the activity of compound **1** against hCA I, producing a homogeneous orange cloud around this moiety (Figure 4B). This result appears to be in agreement with the binding mode predicted for the ligand into hCA I, where the whole aminopyrazole ring of the inhibitor is able to interact with the catalytic site residues of the protein, forming not only H-bonds but also important hydrophobic interactions (Figure 3B). Moreover, the higher importance attributed to the phenyl ring of the ligand by the SHAP analysis for hCA I activity compared to hCA II is consistent with the stronger aromatic/hydrophobic interactions observed for this ligand moiety in hCA I (Figure 3B) compared to hCA II (Figure 3A).

Finally, the SHAP evaluation suggests that only the unsubstituted nitrogen of the ligand pyrazole ring is important for its activity against hCA IX (Figure 4C), while no importance is attributed to this moiety for the activity against hCA XII (Figure 4D). In agreement with these results, only the unsubstituted nitrogen of the ligand pyrazole ring is predicted to establish an H-bond within hCA IX binding site, and no additional interactions are observed for this ligand moiety into the enzyme (Figure 3A), whereas no relevant interactions are formed by the whole aminopyrazole group of compound **1** within hCA XII (Figure 3B). Finally, the either little or negative importance generally attributed to the terminal ethoxycarbonyl moiety of the ligand for its activity against the four hCA isoforms is consistent with the lack of key interactions predicted for this group in any enzyme.

## 3. Discussion

hCAs are involved in numerous physiological processes and have emerged as promising therapeutic targets in a wide range of pathological conditions. However, the development of selective hCA inhibitors remains challenging due to their widespread tissue distribution, and the high sequence and structural similarity among isoforms. In this context, profiling compound activity across multiple hCA isoforms is essential to better understand their selectivity and to guide the identification of modulators with improved therapeutic potential. To tackle this, we developed an ML-based tool for profiling the inhibitory activity of small molecules across a broad panel of isozymes.

To build a reliable predictive framework, we first curated extensive experimental datasets of inhibitory activities against the four hCA isoforms of interest: hCA I, II, IX, and XII. The latter two isozymes are membrane-bound enzymes overexpressed in hypoxic tumors, where they play a direct role in the proliferation and invasiveness of cancerous cells. In contrast, hCA I and hCA II are cytoplasmic forms involved in physiological processes. Nonetheless, both isozymes are therapeutic targets for treating edematous conditions, and hCA II is also relevant for managing glaucoma, epilepsy, and neuropathic pain [2]. The data retrieval and curation steps were critical to ensuring data consistency and to guaranteeing the development of robust predictive models. The resulting datasets, containing between 2800 and 5000 compounds per isoform, were then split into training and test sets in an 80/20 ratio to develop and evaluate ML models. Care was taken to ensure that both training and test sets covered the same chemical space, preserving diversity and preventing bias in model evaluation, as illustrated in Figure S1.

Leveraging the curated datasets, we developed 16 ML models for each isoform, for a total of 64 models. To explore the relationship between modeling strategies and predictive performance, and to identify the most effective model configurations, we systematically evaluated four distinct molecular representations (including FPs and physicochemical descriptors) alongside four distinct ML algorithms. All models were optimized via cross-validation to maximize their predictive accuracy and were subsequently evaluated using a comprehensive set of six performance metrics. Overall, the best-performing models exhibited robust classification capabilities, effectively distinguishing active compounds from inactive ones. Notably, Morgan FPs enabled the most efficient pattern recognition, while SVM and RF algorithms showed superior performance for hCA I-II and hCA IX-XII, respectively.

Therefore, the best model for each enzyme was selected to guide an isoform-specific informed vs. pipeline. This approach was designed not only to identify novel hCA inhibitors but also to integrate insights from the literature to prioritize compounds with known activity against other biological targets. By selecting molecules that had already been explored beyond CAs, we aimed to uncover potential off-target effects and/or assess their suitability for polypharmacological applications, expanding their therapeutic potential. The predicted active compounds resulting from the vs. were then subjected to a multi-step filtering process based on medicinal chemistry and toxicity criteria to ensure the selection of a focused set of high-quality, synthetically feasible, and pharmacologically relevant candidates. Cross-referencing the results obtained by the four vs. pipelines ultimately led to the selection of 12 compounds that were predicted to have promising inhibitory activity profiles, which were subsequently tested in vitro.

Experimental assays confirmed the identification of multiple nanomolar and sub-micromolar inhibitors, in some cases endowed with selectivity for hCA II isoform. Molecular modeling studies enabled the prediction of the potential binding mode of compound **1** within the catalytic sites of the four hCA isoforms, which helped rationalizing the selectivity profile of the newly identified ligand. Specifically, compound **1** was found to engage a conserved interaction pattern via its sulfonamide group across all isoforms, while isoform-specific contacts formed by its aminopyrazole and phenyl moieties explained the observed selectivity. The strongest interaction network was predicted for hCA II, in line with the measured nanomolar potency, whereas reduced interactions with the other isoforms accounted for the progressively lower activity. These results support the structural rationale behind ligand selectivity and further reinforce the predictive value of our integrated modeling approach. Furthermore, these findings validated the reliability of our SHAP-based feature contribution analysis in interpreting ML predictions and identifying the key ligand moieties primarily responsible for their activity. Among various computational methods aimed at relating molecular structure to biological activity [39,40], our reverse-mapping approach specifically focuses on explaining the internal decision-making process of each trained classification model. This provides two key advantages: first, it offers an interpretable rationale behind each specific prediction, overcoming the “black box” nature of many ML models; second, it confirms that the model has effectively captured meaningful structure–activity relationships implicitly present in the training data, as molecular regions deemed important by the model were also predicted to be biologically relevant by orthogonal molecular modeling evaluations. Together, these strengths enhance both the predictive power and biological interpretability of the proposed approach, supporting its use for hypothesis generation and rational ligand design.

Despite the success of our computational pipeline, recognizing its methodological boundaries is critical to understanding its current scope and the potential areas for refinement. First, the study focused on four CA isoforms (hCA I, II, IX, and XII), which are among the most therapeutically relevant and best characterized ones. This selection was driven by the availability of sufficient high-quality data necessary to support robust ML modeling. However, several other isoforms remain underrepresented due to the lack of comprehensive bioactivity datasets, limiting the current platform’s applicability across the full hCA family. Second, the majority of available data on active compounds pertains to primary sulfonamides, a chemotype well known for CA inhibition. As a result, there is a relative scarcity of data concerning active compounds lacking this functional group, which may limit the models’ ability to detect structurally diverse inhibitors beyond this dominant chemical class.

Although the limited availability of data for a broader panel of isoforms and the predominance of primary sulfonamides among active compounds pose inherent challenges, the models developed in this study demonstrated high reliability and predictive robustness. The results obtained, together with the overall hit rate of 77% yielded by the comparison between predictions and experimental data, confirm the effectiveness of the proposed approach for hit identification and activity profiling, paving the way for future optimization studies and expanded therapeutic strategies.

## 4. Materials and Methods

### 4.1. Modeling Datasets

From the ChEMBL 33 database [17], all the compounds with reported inhibitory activity against the targeted human isoforms, namely hCA I, hCA II, hCA IX, and hCA XII, were retrieved (12,876, 15,463, 8007, and 6058 compounds, respectively). Each isoform was treated independently, resulting in the study being structured into four distinct and parallel workflows. The compounds collected for each isozyme were subjected to a filtering procedure which included several sequential steps. Precisely, in the first step, only compounds meeting the following criteria were retained: (1) inhibitory activity reported as *K*_i_ with standard relation type corresponding to “=”, which indicates a certain activity label; (2) molecular weight ≤ 800 Da; (3) number of heavy atoms ≥ 15; (4) containing only atoms from the list [H, B, C, N, O, F, P, S, Cl, Br, I]. To guarantee consistency in assay conditions and to minimize potential biases introduced by inter-laboratory variability, only molecules tested by C.T. Supuran and co-workers (which represented the vast majority of the data collected) were retained. This filtering step was applied at the very beginning of the data processing pipeline, before any classification of compounds into active or inactive, thereby ensuring uniformity in terms of activity measurements and overall data quality. After this filtering procedure, 6608 compounds were retained for CA I, 7044 for CA II, 4794 for CA IX, and 3720 for CA XII. Then, a structure refinement protocol based on the OpenEye chemistry toolkit [41] was applied to remove salts and assess the structural integrity of molecules. Considering that duplicated instances are widely present in databases due to repeated measurements of the same compound or variations in compound representations (e.g., different identifiers for the same chemical structure), a dedicated data curation protocol was implemented to ensure the retention of only unique compounds, and discrepancies in activity values were resolved through a filtering process designed to maintain dataset consistency. For duplicates with three or more reported *K*_i_ values, any value deviating by more than 25% from the calculated mean was excluded, and the mean *K*_i_ was recalculated and assigned as the final potency value. When only two *K*_i_ values were reported instead, the mean was used only if both values deviated by less than 25% from their mean; otherwise, both values were discarded. However, it is important to notice that the ML models developed in this study do not rely on SMILES (Simplified Molecular-Input Line-Entry System) representations but rather on molecular representations. This raises the possibility that two different compounds with different activity values appear to be identical at the molecular representation level (e.g., enantiomers), leading to the model receiving the same molecular representation with different class labels. To tackle this potential issue and strengthen the curation procedure, an additional filter based on molecular representations was applied. To minimize biases arising from reliance on a single perspective, we employed all four representations adopted in this study—Morgan FPs, PubChem FPs, RDKit FPs, and RDKit descriptors, detailed in subsequent sections—thereby ensuring a comprehensive depiction of each compound. Specifically, for each compound pair, the Tanimoto coefficients between the three FP pairs and the Euclidean distance between the descriptor representations were calculated [42], and compounds that appeared identical across all four representations were identified as duplicates. Subsequently, their associated activities were handled similarly to what was previously done. As a result of the duplicate removal procedure, the datasets comprised 5088, 5232, 4130, and 3329 molecules for hCA I, hCA II, hCA IX, and hCA XII, respectively. Upon the obtainment of curated datasets of unique compounds with reliable activity annotations, the distribution of activity values (previously converted in p*K*_i_) was analyzed for each targeted isozyme, and a p*K_i_* value identified at the median of this distribution was set as a threshold for binary classification. To mitigate the impact of a strict classification boundary, all the compounds with a reported p*K*_i_ value within the interval of ±0.25 around the selected cutoff were excluded from the respective dataset. The specific threshold selected for each isoform is reported in Table 3, whereas the activity distribution plot for each dataset is provided in the Supporting Information (Figure S2).

To enhance the datasets and better replicate a realistic activity distribution, each set was enriched with compounds classified as *inactive* based on their reported activity. These compounds were retrieved from the ChEMBL 33 database as those whose activity is expressed as *K*_i_ with a standard relation type corresponding to “>”. During the first stage of data processing, these compounds were excluded because their activity could not be associated with a precise numerical value, precluding their classification at that time. However, once a classification cutoff was established, it became possible to confidently assign these compounds to the inactive class if their reported *K*_i_ value exceeded the threshold. Following this reassignment, these compounds underwent the same data curation process as described previously and were subsequently integrated into the respective datasets. A control procedure ensured the uniqueness of compounds in the resulting integrated datasets. The final labeled datasets were ultimately used to create training and test sets for the corresponding models. Specifically, to maintain the same distribution of active/inactive compounds across each training/test set pair, we randomly selected 80% of the active and 80% of the inactive compounds for the training set, with the remaining 20% of each class forming the isoform-specific hold-out test set. The number of compounds in the four datasets is reported in Table 4.

### 4.2. Molecular Representations

Training an ML model with molecular data involves translating molecular structures into mathematical forms, known as *molecular representations*. These representations capture key molecular features and enable the model to identify patterns that inform learning and drive predictions effectively. In the present study, we used four different molecular representations: three distinct types of molecular FPs (Morgan, RDKit, PubChem) and RDKit descriptors. Both FPs and molecular descriptors are commonly used molecular representations, with their difference lying in that FPs encode structural features as binary arrays, whereas descriptors quantify various physiochemical properties in continuous-number vectors. All the molecular representations were created starting from the SMILES string of the collected compounds.

*Morgan FPs.* They are properly defined as Extended-Connectivity Fingerprints (ECFPs) based on the Morgan algorithm, and for each compound, they were computed using the corresponding implementation of RDKit setting a fixed length of 2048 bits [43]. This representation is derived by considering the local environment around each heavy atom within a specified radius (two bonds for ECFP4 herein used). The information about the atom’s neighbors and their bond types is then encoded into a unique number using a hashing technique, which allows ECFPs to create a compact representation that reflects the structural context of atoms within the molecule.

*RDKit FPs.* In this case, the molecule is split into fragments of different sizes, referred to as subgraphs, with each of them representing a connectivity path within the molecular structure [43]. Each subgraph is then subjected to a hash-based mapping which links it to a specific bit ID. For consistency with Morgan FPs, the RDKit FPs were computed using the implementation provided by the RDKit library, with a fixed length of 2048 bits.

*PubChem FPs.* They consist of 881 bits, where each bit is set to 1 or 0 depending on the presence or absence of specific structural motifs or substructures within the molecule [44].

*RDKit descriptors.* This computational representation is used to encode key structural, chemical, and physicochemical properties of molecules [43]. By exploiting specific functions provided by the RDKit library, the values associated with 208 molecular attributes are derived for each compound, stored as a continuous vector, and normalized. Specifically, normalization was performed using a MinMax scaling approach implemented in Scikit-learn python library version 1.1.1 [19]. This approach scales each feature individually to a fixed range between 0 and 1 while preserving the original distribution shape. Ensuring that all descriptor values are on the same scale is particularly beneficial for ML algorithms, which are sensitive to feature magnitude.

### 4.3. Machine Learning Algorithms

The development of ML models, comprising their implementation and hyperparameters tuning, was executed using the Scikit-learn python library [19]. All models rely on the supervised learning paradigm and are designed to differentiate compounds exhibiting inhibitory activity against the targeted hCA isoforms, individually considered, to those inactive in this regard. With this aim, four different ML algorithms were employed to develop binary classifier models: SVM, RF, KNN, and GP. All models generate a probability score as an output, which represents the likelihood of a compound being active according to the model. Furthermore, each ML architecture is characterized by a set of configuration parameters, referred to as *hyperparameters*. The main hyperparameters considered for each algorithm are reported in this section, whereas for more details about the hyperparameter tuning process, we refer the reader to Section 4.5.

*SVM.* This algorithm operates by projecting input data into a high-dimensional feature space where a hyperplane to linearly separate data points belonging to different classes can be identified [45]. Mapping between the input space and the feature space is achieved through the use of different kernel functions, which enable the model to handle problems of varying complexity. Therefore, the kernel is an important hyperparameter to optimize. This study evaluated several of the most popular kernel functions, including linear, polynomial, RBF, sigmoid, and Tanimoto [46]. While the linear kernel performs a simple dot product, polynomial, RBF, and sigmoid kernels introduce greater non-linearity, allowing the model to capture more complex relationships. The sigmoid kernel, specifically, is based on the hyperbolic tangent and is inspired by neural networks, where a similar transformation is commonly used as an activation function. Both the RBF and Tanimoto kernels are proximity-based. They allow the model to focus more on local patterns, thereby providing greater flexibility in shaping the decision boundary according to the data distribution. This makes them particularly effective in scenarios where class separation is highly non-linear or chemically subtle. To avoid the definition of a decision boundary excessively calibrated on training data, a condition which would lead to overfitting, the regularization hyperparameter *C* can be used to weigh the impact of erroneous predictions during training.

*RF.* This widely used algorithm leverages the combination of multiple weak predictors, known as *decision trees*, to create a robust ensemble model [47]. During training, each tree receives a different subset of training data, which is sampled with replacement, and grows independently without any influence from the other trees. This ensures that each predictor can develop focusing on different characteristics of input data, leading to a more diverse set of decision paths. The outcome of the model is ultimately derived by aggregating the predictions from each individual tree using a majority voting criterion. In this case, the main hyperparameters that can affect the model performance are represented by the number of decision trees and the maximum number of features that a decision tree can use to differentiate the samples.

*KNN.* This algorithm is computationally simple in its core principles, as it relies on a straightforward comparison between the query point and the training instances [48]. Specifically, it computes the distance between the query and each training point, with the subsequent identification of the *k* closest neighbors to the query instance. The model prediction is then determined by the most frequent label among the identified neighbors. For the KNN algorithm, two hyperparameters are of major importance: (a) the number of neighbors to consider for the prediction; (b) whether to weigh neighbors based on their distance from the sample, giving closer neighbors greater influence.

*GP.* This ML algorithm, similarly to SVM, leverages kernels for mapping data into a higher-dimensional space [49]. Therefore, also in GP, the kernel is an important hyperparameter to optimize. The kernels evaluated in this study include DotProduct, RBF, Matern, WhiteKernel, and RationalQuadratic. The DotProduct kernel is well suited for relatively simple data, serving as a baseline kernel. The RBF kernel, similarly to its role in SVM, is proximity-based. It assumes that nearby points are highly correlated, with correlation decreasing as distance increases. The Matern kernel generalizes the RBF by introducing a parameter that controls the smoothness of the function. The RationalQuadratic kernel combines multiple RBF kernels, providing greater flexibility to model functions with varying degrees of smoothness. Finally, the WhiteKernel allows the model to account for noise present in the data, improving robustness. However, unlike SVM, GP adopts a probabilistic approach. Specifically, GP is a probabilistic, non-linear algorithm that leverages Gaussian processes to make inferences about data points. In the classifier case, it defines a probability function that describes the data distribution and allows for the computation of the likelihood that a sample belongs to a specific class.

### 4.4. Performance and Similarity Metrics

Throughout the workflow herein proposed, we used a total of six statistical metrics to evaluate the binary classifier models and assess their performance: MCC, Precision, Recall, Accuracy, Specificity, and NPV [20]. Their formulas are reported below:

(1) $$MCC=\frac{\left(TP\;\cdot \;TN\right)\;-\;\left(FP\;\cdot \;FN\right)}{\sqrt{\left(TP\;+\;FP\right)\left(TP\;+\;FN\right)\left(TN\;+\;FP\right)\left(TN\;+\;FN\right)}}\;$$

(2) $$Precision=\frac{\mathrm{TP}}{\mathrm{TP}\;+\;\mathrm{FP}}$$

(3) $$Recall=\frac{TP}{TP\;+\;FN}$$

(4) $$Accuracy=\frac{TP\;+\;TN}{TP\;+\;TN\;+\;FP\;+\;FN}$$

(5) $$Specificity=\frac{TN}{TN\;+\;FP}$$

(6) $$NPV=\frac{TN}{TN\;+\;FN}$$

TP (true positives) and TN (true negatives) represent the number of active and inactive compounds, respectively, correctly identified by the model. FP (false positives) refers to the number of inactive compounds incorrectly predicted as active, whereas FN (false negatives) refers to the number of active compounds mistakenly classified as inactive. Precision and NPV are ratios of correctly predicted instances for a specific class on all predictions of that class, with the distinction that Precision pertains to positive instances, while NPV applies to negative ones. A similar parallelism can be established between Recall and Specificity, in the sense that they both reflect the ratio of correctly predicted instances on the ground truth number of instances in that class, but Recall refers to positive instances whereas Specificity regards negative ones. Accuracy, on the other hand, is a general metric that calculates the rate of correct predictions across all instances, considering both positive and negative predictions. All the aforementioned measurements range from 0 to 1, with 1 being the best achievable score that occurs when all the predictions considered are correct. It is worth mentioning that Accuracy includes all four possible outcomes of the confusion matrix (TP, TN, FP, FN), but it can be misleading particularly in scenarios of imbalanced datasets. In such cases, a high Accuracy score can be achieved by a model lacking effective learning that simply predicts the majority class.

To address this while still providing a measurement that considers all the possible outcomes, we integrated MCC into our evaluation pipeline as a more robust and unbiased metric. Contrarily to the previous metrics, MCC values span from −1 to 1: −1 indicates completely incorrect predictions, 0 signifies random outcomes, and 1 represents the optimum scenario of all predictions being correct. To compare compounds and quantitatively assess their analogy, the Tanimoto coefficient was computed using the RDKit package [43]. Tanimoto index is a widely used distance metric in cheminformatics to quantify the similarity between two molecules and provides a score that reaches 1 when the two molecular representations compared match exactly. Tanimoto coefficient can be calculated from the binary representations of the chosen compounds according to the following formula:

(7) $$tn=\frac{j}{k+m-j}$$

where *j* is the number of bits set to 1 (*on* bits) in both fingerprints, whereas *k* and *m* are the number of *on* bits in the fingerprint of the first and second molecule, respectively. In cases of continuous value representation instead, the Euclidean distance [42] was used to assess the similarity between compounds.

### 4.5. Models Development and Cross Validation

By combining 4 molecular representations with 4 ML algorithms, 16 ML models were developed for each targeted hCA isoform, for a total of 64 predictive models. Each model was initially subjected to a hyperparameter optimization procedure to identify the setup that maximizes its predictive performance. For this purpose, the exhaustive Grid Search cross-validation method implemented in the Scikit-learn python library was used [19]. This approach systematically tests all possible combinations of specified hyperparameters’ values through a 5-fold stratified cross-validation and assigns a performance score to each combination. For this study, MCC was chosen as the scoring metric. Once the optimal hyperparameters were determined, the models underwent an additional round of evaluation using a 10-fold cross-validation to ensure a thorough performance assessment across diverse data partitions. In each CV cycle, an 80/20 train/validation random split was applied to the isoform-specific training set, and the train split was used to build a control model ultimately tested on the validation set. Upon this robustness evaluation, the final models were developed using the optimal hyperparameter configuration identified. They were trained on the full isoform-specific training dataset and ultimately tested on the isoform-specific hold-out test set to assess their performance on unseen data. For each isoform, the best model was identified and selected to proceed with subsequent stages of the study. These models were also made available as a free tool on our GitHub repository (https://github.com/MMVSL/CAProfiler, accessed on 16 May 2025).

### 4.6. Virtual Screening Dataset and Compounds Selection Criteria

The vs. library was obtained as a curated subset of the ChEMBL 33 database (accessed in November 2023). Specifically, from the whole ChEMBL 33 database, we removed all compounds with activity against carbonic anhydrases for any organisms. The remaining 2′361′912 molecules were subjected to an analogous curation protocol to the one employed for constructing the training models (see *Modeling Datasets*). As a result, we obtained a refined library of around 2 million molecules to screen for the potential inhibitory activity against hCA I, hCA II, hCA IX, and hCA XII. Predicted active compounds were then pooled together and progressively filtered through a series of selection steps based on commercial availability, chemical structure, drug-likeness, toxicity, and structural novelty criteria. Initially, only commercially available molecules were retained, and Pan-Assay Interference (PAINS) [21] compounds were excluded using MolBook UNIPI [22]. The remaining compounds were then filtered for drug-likeness and synthetic feasibility using Lipinski’s Rule of Five and Veber’s Rule. To further refine the dataset, molecules containing chemically undesirable or toxicophoric substructures were discarded using several structural alert filters, including Brenk, Bristol-Myers Squibb (BMS), Inpharmatica, and Novartis NIBR rules [23,24,25]. An additional toxicity screening was conducted with the VenomPred 2.0 platform [26], focusing on endpoints relevant to hCA inhibitor development, namely eye irritation, acute oral toxicity, carcinogenicity, and hepatotoxicity. Lastly, to ensure structural novelty and encourage chemical diversity, literature mining was used to retain molecules previously reported as active on targets other than carbonic anhydrases, and compounds highly similar to those in the training sets (Tanimoto similarity greater than 0.80 calculated using PubChem FPs) were excluded.

### 4.7. Carbonic Anhydrases In Vitro Inhibition Test

The inhibitory activity of the 12 purchased compounds was tested in vitro using a CO_2_ hydrase stopped-flow assay using acetazolamide (AAZ) as a reference drug. An SX.18 MV-R Applied Photophysics stopped-flow instrument was utilized [50]. A 0.2 mM phenol red was used as an indicator, operating at its maximum absorbance of 557 nm, with 10 mM Hepes (pH 7.4) as a buffer. The ionic strength was maintained at a constant using 0.1 M Na_2_SO_4_ or NaClO_4_, as these anions are non-inhibitory at the concentration employed. The CA-catalyzed CO_2_ hydration reaction was monitored for a period of 5–10 s. A saturated aqueous CO_2_ solution at 25 °C, with [CO_2_] of 17 mM, was used as the substrates. Inhibitor stock solutions were prepared at a concentration of 10 mM in 1:1, *v*/*v* DMSO/water mixture and were subsequently diluted up to 0.01 nM in the assay buffer. To determine the inhibition constant (*K*_i_), at least seven different inhibitor concentrations were tested. The enzyme and the inhibitor were pre-incubated for 15 min to allow the complete formation of the enzyme-inhibitor complex. Each inhibitor concentration was tested in triplicate, and the values reported throughout the study represent the mean of these replicates. The inhibition constants were obtained by non-linear least-squares fitting using PRISM3 and the Cheng–Prusoff equation, as previously reported [51]. All CA isoforms were recombinant proteins expressed in-house in *Escherichia coli*, as described in previous studies from the corresponding synthetic genes. Their concentrations in the assay system were as follows: [CA I] = 11.7 nM; [CA II] = 6.8 nM; [CA IX] = 8.2 nM; [CA XII] = 10.7 nM [52]. These enzyme concentrations were measured by titration with ethoxzolamide and spectrophotometrically for CA I and II.

### 4.8. Molecular Dynamics Simulations

The crystal structures of hCA I (PDB code 1AZM), hCA II (PDB code 2AW1), hCA IX (PDB code 3IAI), and hCA XII (PDB code 1JD0) were taken from the Protein Data Bank [53] and used for the MD simulations, which were performed using AMBER, version 20 [54], employing the ff14SB force field. The proteins were placed in a parallelepiped water box, by using TIP3P explicit solvent model, and solvated with a 15.0 Å water cap. Sodium ions were added as counterions to neutralize the solvated systems. Before each MD simulation, the systems were energy minimized using a two-stage protocol. In the first stage, 5000 steps of steepest descent (SD) followed by conjugate gradient (CG) algorithms were performed for the exclusive minimization of the solvent, since a harmonic potential of 100 kcal/(mol·Å^2^) was applied to all solute atoms. In the second stage, 5000 additional steps of SD/CG were used to minimize the whole systems, until a convergence of 0.05 kcal/(mol·Å^2^), imposing a harmonic constraint of 10 kcal/(mol·Å^2^) only on the protein α carbons. The minimized systems were used as starting conformations for the MD simulations, which were performed using Particle Mesh Ewald (PME) electrostatics, periodic boundary conditions, and a cutoff of 10 Å for the non-bonded interactions. SHAKE algorithm was employed to keep all bonds involving hydrogen atoms rigid. The energy minimized systems were then subjected to a heating MD stage corresponding to 1 ns of constant volume periodic boundary MD, during which the temperature was raised from 0 to 300 K. Subsequently, a 5 ns equilibration stage of constant pressure MD was performed using the Langevin thermostat to maintain the temperature at the constant value of 300 K. During such MD stages, the simulations were performed using a time step of 2.0 fs. Finally, a production stage consisting of 100 ns of constant pressure MD simulation was performed using the hydrogen mass repartition (HMR) scheme with a time step of 4.0 fs, since this technique proved to be useful to reduce the simulation time while maintaining an unbiased MD protocol [55]. From each MD simulation, a total of 100 MD snapshots (one every nanosecond of simulation) were saved using cpptraj program [56], implemented in AMBER 20, to be used for the ensemble docking studies.

### 4.9. Ensemble Docking

The structure of compound **1** was built using the last version of the software MolBook UNIPI [22]. The software GOLD 5.1 [57] with PLP fitness function was employed for molecular docking, as previously performed [11]. The binding cavity used for the docking calculations was defined in order to include all residues which stayed within 15 Å from the center of the co-crystallized ligand in the reference X-ray complex. In all docking studies, the possibility for the ligand to flip ring corners was enabled, whereas the “allow early termination” option was deactivated. The ligand was docked into 100 different conformations for each of the four hCA isoforms, which were derived from the corresponding MD simulations, for a total of 400 different docking studies. In each docking calculation, the ligand was subjected to 100 genetic algorithm runs, and GOLD defaults were used for all other settings. The different docking poses were clustered based on the ligand disposition within the protein binding site, employing an RMS tolerance of 2.0 Å. The best docked conformations were selected and subjected to a further energy minimization in explicit water environment following the same system preparation and double step minimization protocol employed prior to the MD simulations described in the previous paragraph.

### 4.10. Feature Contributions and Importance Mapping

The contributions to model predictions were assessed using the Shapley value framework. Originally developed to evaluate the influence of individual players within a cooperative setting, the SHAP (SHapley Additive exPlanations) method [38] estimates the impact of each feature based on its marginal contribution to the overall model output. In the context of binary classification, as applied in this study, SHAP values were calculated with signed importance: a positive value indicates a contribution toward the prediction of activity, whereas a negative value reflects a contribution toward the prediction of inactivity. Due to the model-dependent nature of standard SHAP implementations, we opted for the model-agnostic Permutation SHAP approach, available in the SHAP Python library. To provide a consistent visualization of the SHAP-derived results at the atomic level, feature contributions were mapped back to individual atoms using a retro-mapping strategy. Specifically, for Morgan fingerprints, atom-level attribution was achieved by leveraging RDKit’s built-in functionalities to identify the atoms associated with each active fingerprint bit. A feature-weighting scheme was then applied to assign a representative score to each atom. Finally, atomic contributions were visualized using RDKit’s mapping functions.

## 5. Conclusions

hCAs represent a complex and therapeutically relevant enzyme family, whose selective modulation remains a key challenge in drug discovery. In this study, we developed a robust ML-based tool for the prediction and profiling of inhibitory activity of small-molecules across multiple human CA isoforms, namely hCA I, II, IX, and XII. Notably, the developed ML tool addresses a critical gap in drug discovery by being the first in this context, to the best of our knowledge, to apply ML across such a significant spectrum of isoforms. By combining curated bioactivity data and diverse modeling strategies, we developed predictive models capable of effectively identifying novel inhibitors of the considered isoforms. The reliability of the proposed approach was confirmed through experimental validation and further supported by structure-based modeling studies, as well as interpretable feature contribution analysis. To foster transparency, reproducibility, and broad dissemination, the entire ML tool has been made publicly accessible through a GitHub repository. Moreover, we plan to integrate the tool into MolBook [22], a comprehensive software solution for chemical data management. By incorporating our tool into MolBook, we aim to provide a powerful and user-friendly tool that can be accessed by the entire scientific community without the need for computational expertise. This integration will further enhance the scientific impact of the proposed framework and broaden its applicability across diverse research domains.

## Figures and Tables

**Table 1:** Performance of the top-scoring model for each hCA isoform considered on the isoform-specific hold-out test set.

	hCA I	hCA II	hCA IX	hCA XII
**Model**	SVM—Morgan FPs	SVM—Morgan FPs	RF—Morgan FPs	RF—Morgan FPs
**MCC**	0.79	0.79	0.73	0.73
**Accuracy**	0.90	0.90	0.86	0.86
**Precision**	0.87	0.87	0.85	0.85
**Recall**	0.89	0.89	0.85	0.88
**Specificity**	0.91	0.90	0.88	0.85
**NPV**	0.92	0.92	0.88	0.88

**Table 2:** Structure and experimental hCA inhibitory activity profile of the tested compounds using acetazolamide (AAZ) as a reference inhibitor.

Cpds.	Structure	Experimental *K*_i_ (nM)			
		hCA I	hCA II	hCA IX	hCA XII
**1**	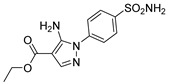	7.3	1.4	67.7	716.5
**2**	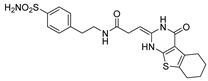	1023.0	11.6	713.1	30.6
**3**	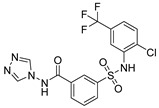	>100,000	>100,000	>100,000	>100,000
**4**	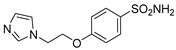	36.2	6.8	2523.8	603.0
**5**	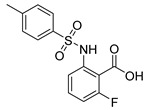	>100,000	>100,000	>100,000	>100,000
**6**	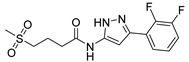	>100,000	7050.3	>100,000	>100,000
**7**	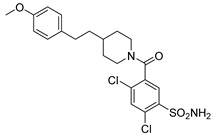	2858.1	9.8	6826.7	350.9
**8**	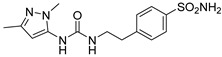	1204.8	23.9	715.4	832.5
**9**	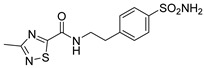	212.6	443.6	728.5	568.4
**10**	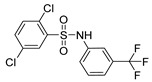	31,589	>100,000	>100,000	>100,000
**11**	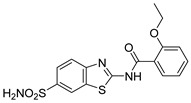	665.6	16.4	73.7	49.4
**12**	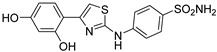	1076.3	151.4	313.2	648.1
**AAZ**		250.0	12.1	25.3	5.7

**Table 3:** Values of p*K*_i_ selected as cutoff for binary classification. Compounds with reported activity falling within the interval were removed from each dataset. The thresholds were identified for each dataset according to the activity distribution.

hCA Isoform	p*K_i_* Threshold Value (±Interval)
hCA I	6.30 (±0.25)
hCA II	7.30 (±0.25)
hCA IX	7.30 (±0.25)
hCA XII	7.45 (±0.25)

**Table 4:** Composition in terms of active/inactive compounds for training and test sets of each targeted isoform.

Datasets hCA I			
	**Active**	**Inactive**	**Total**
**Training**	1618	2278	3896
**Test**	405	570	975
**Datasets hCA II**			
	**Active**	**Inactive**	**Total**
**Training**	1682	2319	4001
**Test**	421	580	1001
**Datasets hCA IX**			
	**Active**	**Inactive**	**Total**
**Training**	1208	1512	2720
**Test**	302	379	681
**Datasets hCA I**			
	**Active**	**Inactive**	**Total**
**Training**	1104	1166	2270
**Test**	277	292	569

## Data Availability

The data that support the findings of this study are available at the following link: https://github.com/MMVSL/CAProfiler (accessed on 16 May 2025).

## Supplementary Materials

The following supporting information can be downloaded at: https://www.mdpi.com/article/10.3390/ph18071007/s1, Figure S1: Overlaid t-SNE projection of training and test tests for each isoform; Figure S2: Distribution plots of p*K*_i_ values in curated datasets for threshold identification; Table S1: Cross validation scores for each model; Table S2: Comparison between predictions and experimental activity for the tested compounds.
