# Supplementary material for: A Machine Learning Platform for Isoform-Specific Identification and Profiling of Human Carbonic Anhydrase Inhibitors
Source: Pharmaceuticals (Basel). 2025 Jul 5;18(7):1007. doi: 10.3390/ph18071007 (PMC12300992; doi:10.3390/ph18071007)
Supplement: Supplementary file 1 [file pharmaceuticals-18-01007-s001.zip › pharmaceuticals-3679004-supplementary.pdf]

Supporting Information

A Machine Learning Platform for Isoform-Specific Identification and Profiling of Human Carbonic Anhydrase Inhibitors

Lisa Piazza[1], Miriana Di Stefano[1], Clarissa Poles[2,3], Giulia Bononi[1], Giulio Poli[1], Gioele Renzi[4], Salvatore Galati[1], Antonio Giordano[5], Marco Macchia[1], Fabrizio Carta[4], Claudiu T. Supuran[4], and Tiziano Tuccinardi[1,6,*]

[1] Department of Pharmacy, University of Pisa, Pisa, Italy

[2] Telethon Institute of Genetics and Medicine, Naples, Italy

[3] Genomics and Experimental Medicine Program, Scuola Superiore Meridionale (SSM, School of Advanced Studies), Naples, Italy

[4] Department of Neurofarba, Section of Pharmaceutical and Nutraceutical Sciences, University of Florence, Polo Scientifico, Via U. Schiff 6, Sesto Fiorentino, 50019 Firenze, Italy

[5] Sbarro Institute for Cancer Research and Molecular Medicine, Center for Biotechnology, College of Science and Technology, Temple University, Philadelphia, PA, USA

[6] Consorzio Interuniversitario Nazionale per la Scienza e Tecnologia dei Materiali (INSTM), Firenze, Italy

* Correspondence: tiziano.tuccinardi@unipi.it

Table of Contents

Figure S1 Overlaid t-SNE projection of training and test tests for each isoform S2

Table S1 Cross validation scores for each model S3

Table S2 Comparison between predictions and experimental activity for the tested compounds S4

Figure S2 Distribution plots of pK_i values in curated datasets for threshold identification S5

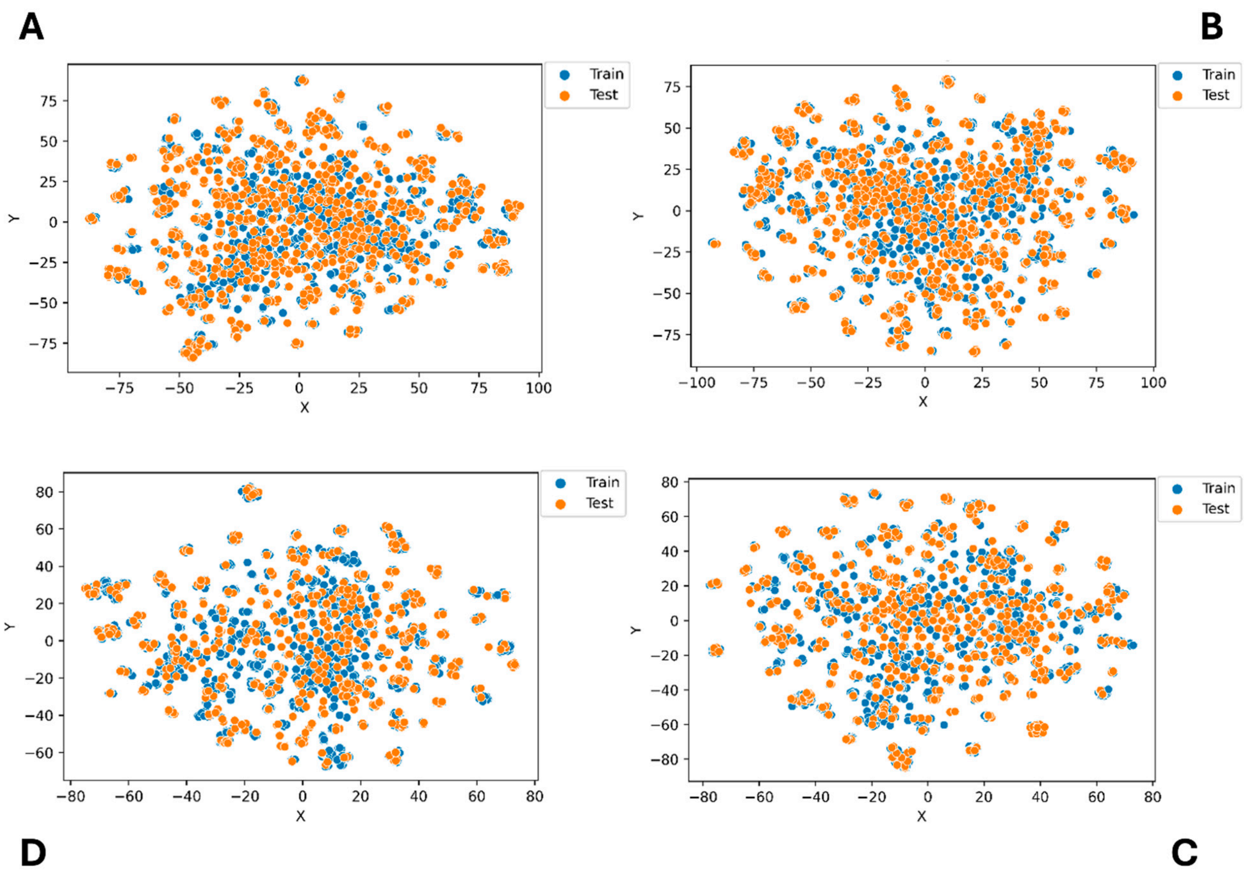

Figure S1. t-SNE superimposed projection of train and test set for each targeted isoform. The t-SNE was obtained for each training/test sets pair using Morgan FPs as the molecular representation, which is the representation that characterizes the top performing models. Data for hCA I, hCA II, hCA IX and hCA XII were labelled with **A, B, C** and **D**, respectively.

Table S1. Performance, evaluated through MCC, achieved by all models during the 10-fold CV. Rows refer to the algorithm characterizing the model and the molecular representation used, columns to the hCA isoform for which the model was developed.

	**hCA I**	**hCA II**	**hCA IX**	**hCA XII**
**SVM - Morgan FPs**	0.75 ± 0.01	0.74 ± 0.01	0.68 ± 0.01	0.65 ± 0.03
**SVM - RDKit FPs**	0.72 ± 0.02	0.74 ± 0.01	0.69 ± 0.01	0.61 ± 0.06
**SVM - PubChem FPs**	0.73 ± 0.02	0.74 ± 0.02	0.67 ± 0.03	0.63 ± 0.01
**SVM - RDKit desc.**	0.72 ± 0.01	0.70 ± 0.01	0.64 ± 0.02	0.56 ± 0.01
**RF - Morgan FPs**	0.76 ± 0.0	0.76 ± 0.02	0.69 ± 0.03	0.64 ± 0.03
**RF - RDKit FPs**	0.74 ± 0.0	0.76 ± 0.0	0.71 ± 0.0	0.66 ± 0.0
**RF - PubChem FPs**	0.74 ± 0.03	0.72 ± 0.02	0.68 ± 0.03	0.65 ± 0.0
**RF - RDKit desc.**	0.76 ± 0.0	0.72 ± 0.0	0.63 ± 0.04	0.58 ± 0.03
**KNN - Morgan FPs**	0.71 ± 0.01	0.71 ± 0.03	0.66 ± 0.02	0.63 ± 0.01
**KNN - RDKit FPs**	0.69 ± 0.02	0.69 ± 0.02	0.64 ± 0.02	0.62 ± 0.03
**KNN - PubChem FPs**	0.66 ± 0.02	0.67 ± 0.02	0.59 ± 0.02	0.58 ± 0.03
**KNN - RDKit desc.**	0.69 ± 0.01	0.68 ± 0.03	0.60 ± 0.03	0.57 ± 0.03
**GP - Morgan FPs**	0.74 ± 0.02	0.72 ± 0.01	0.68 ± 0.02	0.63 ± 0.02
**GP - RDKit FPs**	0.68 ± 0.02	0.70 ± 0.02	0.64 ± 0.01	0.60 ± 0.04
**GP - PubChem FPs**	0.72 ± 0.02	0.73 ± 0.02	0.66 ± 0.03	0.62 ± 0.02
**GP - RDKit desc.**	0.73 ± 0.02	0.70 ± 0.02	0.64 ± 0.03	0.59 ± 0.02

Table S2. Comparison between predicted and experimental activity class for the selected compounds. For each compound the experimental class is reported in bold if in accordance with the prediction. A compound is classified as experimentally active or inactive based on a K_i threshold of 1 μM, while it is considered predicted as active when the probability returned by the model is ≥ 50%.

Compounds	hCA I	hCA II	hCA IX	hCA XII
1	**Active**	**Active**	**Active**	**Active**
2	**Inactive**	Active	**Active**	**Active**
3	**Inactive**	Inactive	**Inactive**	**Inactive**
4	**Active**	**Active**	Inactive	**Active**
5	Inactive	Inactive	**Inactive**	**Inactive**
6	Inactive	**Inactive**	**Inactive**	**Inactive**
7	**Inactive**	**Active**	Inactive	Active
8	Inactive	**Active**	**Active**	**Active**
9	**Active**	**Active**	**Active**	**Active**
10	**Inactive**	Inactive	**Inactive**	**Inactive**
11	**Active**	**Active**	**Active**	**Active**
12	Inactive	**Active**	**Active**	**Active**

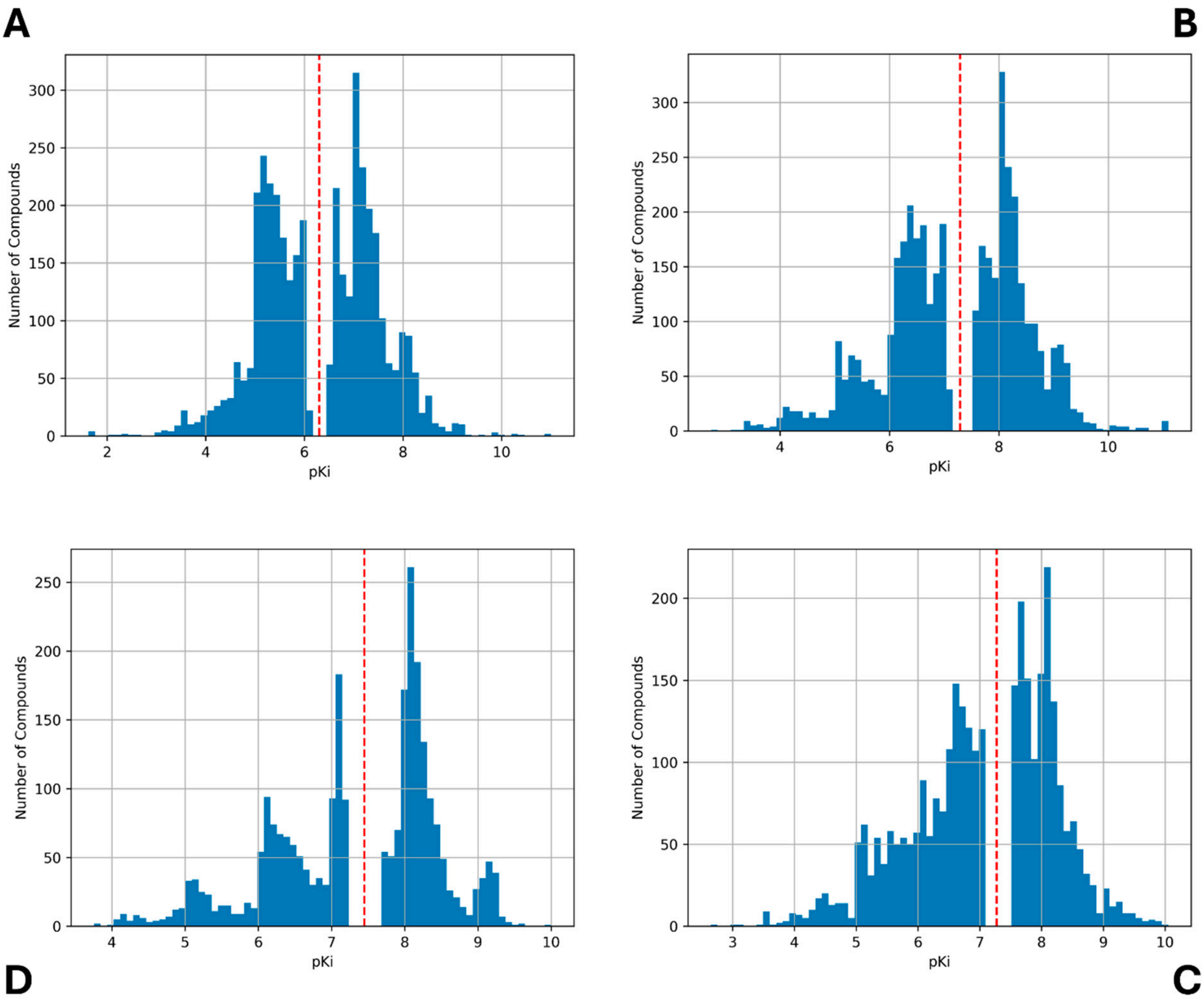

Figure S2. Distribution plots of pK_i values of compounds in curated dataset before a further integration with inactive compounds. These distributions were used to identify the threshold value for binary classification, set at the median of the distribution, and represented in each plot by the red dashed line. Chemicals falling within ±0.25 around the interval were removed, as evidenced in the plots. Data for hCA I, hCA II, hCA IX and hCA XII were labelled with **A, B, C** and **D**, respectively.
